# Polymerase cross-linking spiral reaction (PCLSR) for detection of African swine fever virus (ASFV) in pigs and wild boars

**DOI:** 10.1038/srep42903

**Published:** 2017-02-15

**Authors:** Grzegorz Woźniakowski, Magdalena Frączyk, Andrzej Kowalczyk, Małgorzata Pomorska-Mól, Krzysztof Niemczuk, Zygmunt Pejsak

**Affiliations:** 1 Department of Swine Diseases, National Veterinary Research Institute (NVRI), Partyzantów 57 Avenue, 24-100 Puławy, Poland; 2 Chief Executive, National Veterinary Research Institute (NVRI), Partyzantów 57 Avenue, 24-100 Puławy, Poland

## Abstract

The study reports the development of a polymerase cross-linking spiral reaction (PCLSR) for the detection of African swine fever virus (ASFV) DNA in blood collected from infected pigs and wild boars. The method uses 3 specifically designed primers. Two outer-spiral primers comprising of 3′ sequences complementary to ASFV p72 gene sequence and 5′end sequences complementary to exogenous gene of black widow alpha-latrotoxin as well as additional ASFV specific cross-linking primer. The method is specific exclusively to ASFV DNA without cross-reactions with cDNA of classical swine fever virus (CSFV), porcine reproductive respiratory syndrome (PRRSV) or porcine epidemic diarrhea virus (PEDV). The sensitivity of this technique reached 7.2 × 10^2^ copies per *μ*l^−1^ of plasmid containing p72 gene. The PCLSR was conducted at 65 °C creating cross-linked complex structures. The results of PCLSR were visualized using SYBR Green I dye, gel electrophoresis while the reaction progress was traced using real-time PCR system that resulted in registration of fluorescent curves and melting peaks at 85.3 °C. The developed PCLSR was examined using blood or tissue samples collected from selected 17 ASF cases from infected wild boars and 3 outbreaks in pigs. Further tests have been also conducted using 55 tissue samples from 23 outbreaks and 22 cases. These results showed that PCLSR might be further used for preliminary and cost-effective detection and surveillance of ASFV.

African swine fever (ASF) is currently an issue of great importance for international trade of pigs and pig-origin meat products^1^. ASF is an infectious viral disease of pigs, wild boars and other free-ranging suids^2^,^3^. ASF is also a notifiable disease, due to consequences related to its occurrence in the affected country or region^4^. The main reservoir of ASF and the main transmission source within the territory of Mediterranean climate are soft-ticks belonging to Ornithodoros genus^5^. The main transmission source of ASF within the territory of eastern Europe are wild boars. ASF is caused by the African Swine Fever Virus (ASFV) belonging to *Asfivirus* genus of the *Asfarviridae* family. Taking into account the current status of ASF in Poland it is the country most severely affected, acting as “boundary zone” between infected and uninfected parts of Europe. Considering the current epidemiological situation of Poland, the important role of the National Reference Laboratory (NRL) for diagnosis of ASF should be emphasized. Currently, all ASFV genotypes circulating on the territory of eastern Europe belong to genotype II of the remaining 22 other genotypes^1^,^2^. The laboratory diagnostics of ASF are under strict recommendations of the World Organization for Animal Health (OIE) as well as the European Reference Laboratory (EURL). The recommended diagnostic methods include real-time PCR for detection of ASFV DNA^6^,^7^,^8^ as well as enzyme-linked immunosorbent assay (ELISA)^9^,^10^ and immunoperoxidase test (IPT)^11^,^12^ or the identification and/or confirmation of anti-ASFV antibodies. Two other alternative detection assays including loop-mediated isothermal amplification (LAMP) as well as cross-priming amplification - CPA have been developed^13^,^14^, and showed capable of ASFV detection. Although both tests are sensitive and rapid, they might provide some percentage of false positive results due to interaction of multiple primer pairs^15^,^16^. The presented study aimed to develop a novel polymerase cross-linking spiral reaction (PCLSR) which applies only 3 primers and requires a polymerase with strand-displacement activity including *Bst, Bsm* or *Gsp*SSD. The developed assay shows some similarities with LAMP, (CPA)^15^,^17^, nucleic acid sequence dependent amplification (NASBA)^18^, rolling circle replication (RCR)^19^ and recently polymerase spiral reaction (PSR) described for the first time by Liu *et al*.^20^ then subsequently applied by other scientists for detection of Candida albicans^21^. All these methods have an advantage to overcome the requirement of cycling which is compulsory in case of PCR-based techniques or real-time PCR. However, the mechanism and progress of PCLSR is different and relies on formation of 3 independent prerequisite spiral products. Similarly, to CPA or PSR the final cross-linked products form complex entities which result from target DNA sequence multiplication. The final products detection is mediated after addition of double-DNA binding fluorescent dye including SYBR Green^®^ I. Having the possibility to trace the reaction progress, the developed PCLSR can also be performed using real-time PCR system as a semi-quantitative technique. The results are finally registered as fluorescence of samples containing ASFV DNA under UV light as greenish fluorescence, or the presence of fluorescent curves at a particular cycle of the reaction using real-time PCR system. To the best of our knowledge, this is the first report of development of the PCLSR as an alternative assay for other described isothermal detection methods. The PCLSR presents a simple alternative for real-time PCR techniques recommended by OIE or EURL for ASF diagnosis and hopefully might further be considered as an official method. The PCLSR can also be easily adapted for on-site identification of other human, animal or plant pathogens.

## Results

### Design of PCLSR

**Figure 1:**
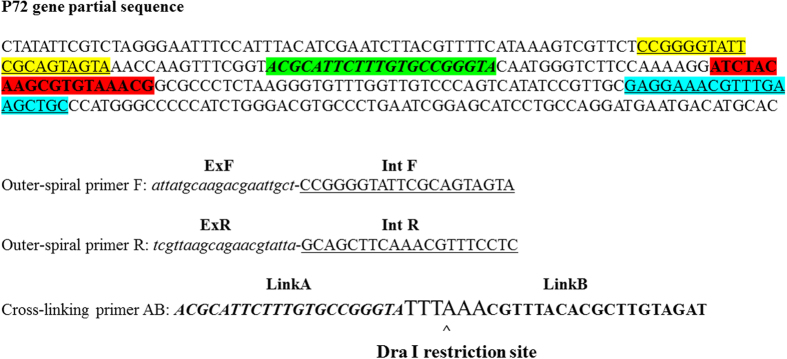
Location and nucleotide sequences of PCLSR primers within the p72 gene of Georgia 2007/1 isolate ASFV (Accession number: FR682468.1). The names of particular regions of outer spiral and cross-linking primers AB are indicated above the color-highlighted sequences. The *DraI* restriction site has been marked.

The PCLSR method relies on isothermal amplification of targeted DNA using DNA polymerase showing strand displacement features. The primer design was similar to those applied in other isothermal methods but included 2 outer-spiral primers with 5′ terminal ExF and ExR sequences reverse complementary to DNA of black widow (*Latrodectus hesperus*) alpha-latrotoxin (position 180–199) (Genbank accession: KF751511.1). The 3′ sequence of outer-spiral primers (IntF and IntR) were complementary to the p72 gene sequence (positions 104980–104999 and 105116–105135) of Georgia 2007/1 strain (Genbank accession: FR682468.1). The cross-linking primer AB (comprising of Link A and Link B) was complementary exclusively to p72 gene sequence of ASFV and included *DraI* restricition site for further confirmation of results specificity (positions 105014–105034 and 105054–105072). The primer location and sequence has been shown in Fig. 1.

### Reaction mechanism

**Figure 2:**
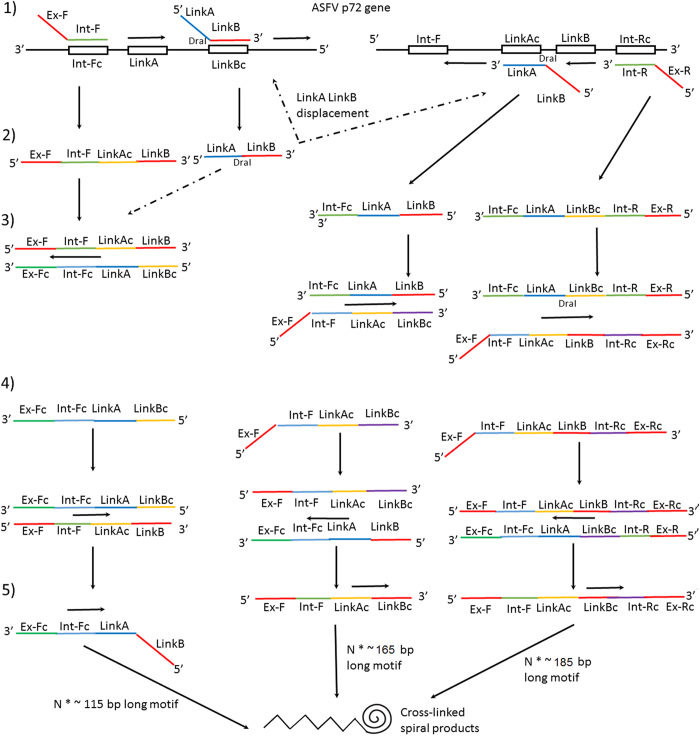
Mechanism of PCLSR. At the initial stage the double structure of DNA is displaced by GspSSD polymerase (OptiGene, Horsham, West Sussex, United Kingdom). The first stage (1) on the left part shows the cross-linking primer is ligating with its LinkB fragment to the targeted sequence of p72 ASFV gene, then the polymerase extends the product from 5′ to the 3′ end of the structure. At the same time the process undergoes on the opposite 3′–5′ strand of p72 gene (right part). The outer primers ligate with their 3′ ends (Inf-F and Int-R) to the complementary external sequences of the targeted region. Amplification results in displacement of the newly synthesized products (left and right structure). Next, during the following stages the complementary strands are synthesized which leads to the target primary sequence multiplication. During the reaction progress three parallel structures with different sizes (115 bp, 165 bp and 185 bp) are synthesized (Structures 3, 4). The remaining 5′ ends of the products, namely ExF and ExR of outer-spiral primers which are reverse-complementary ligate to each other and form complex cross-linked spiral structures (Structure 5).

The mechanism of PCLSR has been presented in Fig. 2. During the initial stage at 65 °C the double structure of DNA is displaced by GspSSD polymerase (OptiGene, Horsham, West Sussex, United Kingdom). The first stage (1) (left part of Fig. 2) shows the cross-linking primer ligating with its LinkB fragment to the targeted sequence of p72 ASFV gene, with the polymerase extending the product from 5′ to the 3′ end of the structure. At the same time, the same process occurs on the opposite 3′–5′ strand of p72 gene (right part of the Fig. 2). The next step is the ligation of the outer primers with their 3′ ends (Int-F and Int-R) to the complementary external sequences of the targeted region (1). Within the stage (2) amplification results in displacement of the newly synthesized products - Fig. 2 left and right structure (2). Next, during the following stages the complementary strands are synthesized and this leads to multiplication of the target primary sequence (3 and 4). During the reaction progress three parallel structures with different size (115 bp, 165 bp and 185 bp) are synthesized (stages 3 and 4). The remaining 5′ ends of the products, namely ExF and ExR of outer-spiral primers which are reverse-complementary ligate to each other and form complex cross-linked spiral structures (stage 5).

### Examination of PCLSR parameters

**Figure 3:**
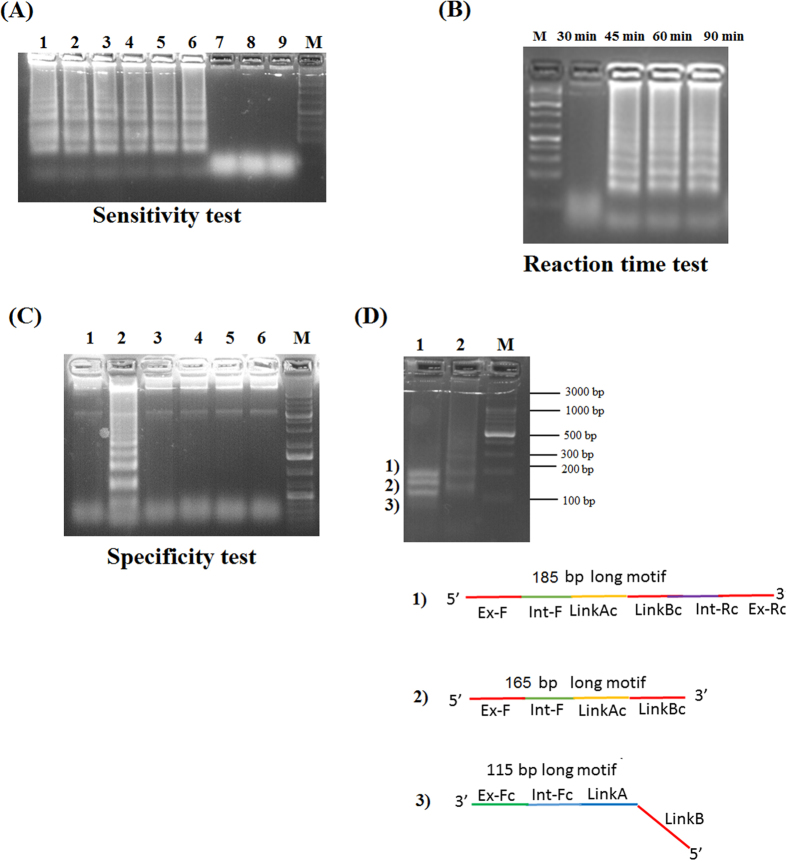
PCLSR parameters evaluation. (**A**) PCLSR sensitivity test. Gel electrophoresis of resulted products. Serial 10-fold dilutions of a standard ASFV plasmid with p72 gene fragment (from 7.2 × 10^7^ – lane #1 copies, to 0.07 copies, *μ*l^−1^ – lane #9). The limit of detection (LoD) reached 7.2 × 10^2^ copies, *μ*l^−1^ (lane #6). (**B**) PCLSR reaction time test. Incubation of reaction mixtures in time from 30 min. up to 90 min. The first presence of specific “ladder-like” products is observed after 45 min. (**C**) Specificity test. 1 – blood collected from a non-infected pig, 2 – positive control standard ASFV plasmid containing a p72 gene fragment (7.2 × 10^7^ copies *μ*l^−1^); 3 – blood collected from a non-infected wild boar; 4 –cDNA extracted from a classical swine fever virus (CSFV) strain Alfort/187; 5 – cDNA extracted from a porcine reproductive and respiratory syndrome virus (PRRS) strain Lelystad (genotype I); 6 – cDNA of field porcine epidemic diarrhea virus (PEDV-NVRI2015). (**D**) Restriction analysis of PCLSR products using *DraI* enzyme (Thermo-scientific, Waltham, Massachusetts, USA). 1 – The digestion of PCLSR products resulted in occurrence of 3 different products sizing 115 bp, 165 bp and 185 bp, respectively. 2 – positive control standard ASFV plasmid containing a p72 gene fragment (7.2 × 10^7^ copies *μ*l^−1^). The structures 1)-3) present 3 obtained products. M – molecular length marker GeneRuler 100 bp DNA Ladder Plus (Thermo-scientific, Waltham, Massachusetts, USA).

**Figure 4:**
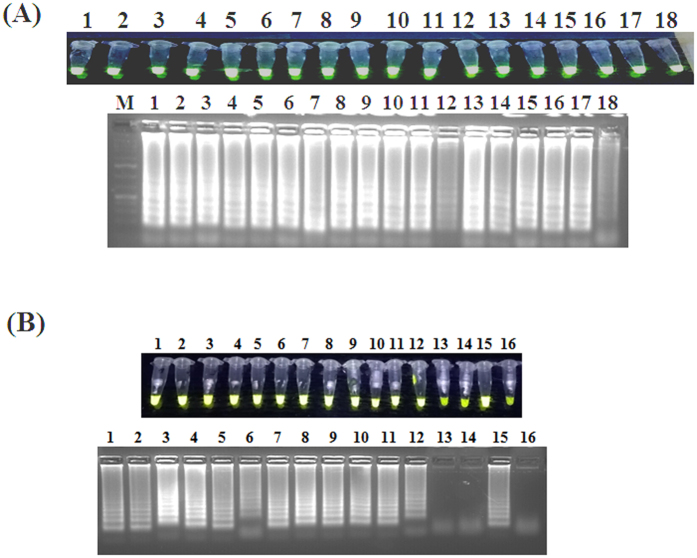
PCLSR evaluation using different ASFV genotypes or strains and matrices. (**A**) Genotype and strain specificity testing. 1 – CV98 strain (genotype I), 2 – BF07 strain (genotype I), 3 – E70 strain (genotype I), 4 – Arm07 strain (genotype II), 5 – LT14/1490 (genotype II), 6 – Ukr12/Zapo (genotype II), 7 – Estonia 1 (genotype II), 8 – Estonia 2 (genotype II), 9 – Estonia 3 (genotype II), 10 – Estonia 4 (genotype II), 11 – Estonia 5 (genotype II), 12 – Estonia 6 (genotype II), 13 – Estonia 7 (genotype II), 14 – Moz64 (genotype V), 15 – Malawi (genotype VIII), 16 – Ken06.Bus (genotype IX), 17 – Ken08K.2/1 (genotype X), 18 – Ug64 (genotype X). (**B**) Testing of matrices from infected animals. 1 – bone marrow diluted 1:10 w/v, 2 – bone marrow concentrated, 3 – blood of pig, 4 – blood of pig (clot), 5 – serum of pig, 6 – blood of wild boar, 7 – spleen, 8 – kidney, 9 – lymph node, 10 – tonsil, 11 – lungs, 12 – muscle fibers, 13 – cDNA extracted from a classical swine fever virus (CSFV) strain Alfort/187; 14 – cDNA extracted from a porcine reproductive and respiratory syndrome virus (PRRS) strain Lelystad (genotype I), 15 – positive control standard ASFV plasmid containing a p72 gene fragment (7.2 × 10^7^ copies *μ*l^−1^), 16 – blood collected from a non-infected pig.

The PCLSR was conducted at 65 °C being the most optimal temperature using a water bath. The sensitivity test presented as the electrophoresis plot in 1.5% agarose gel (Invitrogen) stained with SimplySafe solution (EurX) showed the presence of ladder-like pattern of bands in serial 10-fold dilutions of a standard ASFV plasmid with p72 gene fragment (from 7.2 × 10^7^ copies to 0.07 copies *μ*l^−1^) (Fig. 3A, lane 1–9). The obtained limit of detection (LoD) reached 7.2 × 10^2^ copies *μ*l^−1^ (Fig. 3A, lane 6). The conducted examination of the optimal reaction time showed that the PCLSR was capable of amplifying ASFV DNA in 45 min (Fig. 3B, lane 45 min). Longer amplification time does not result in higher yield of DNA products (Fig. 3B, lanes 60 min–90 min). The specificity test showed PCLSR was specific only for ASFV DNA (Fig. 3C, lane 2). No presence of specific ladder-like products was observed in negative controls represented by DNA extracted from spleen of healthy wild boar, pig as cDNA of classical swine fever virus (CSFV), cDNA of porcine reproductive respiratory syndrome virus (PRRSV) or porcine epidemic diarrhea virus (PEDV) (Fig. 3C, lanes 1, 3–6). The performed digestion of PCLSR products (Fig. 3D, lane 2) using *DraI* enzyme showed the presence of 3 distinct products of 115, 165 and 185 bp long (Fig. 3D, lane 1). These results indicate the presence of 3 prerequisite sequences for further amplification of cross-linked spiral products. The obtained bands were cut off from the gel, purified and sequenced. All three bands showed 100% identity to the p72 gene of Georgia 2007/1 strain of ASFV (data not shown). The products of PCLSR were detected using 3 methods: by observation of greenish fluorescence under UV light in ASFV positive samples after addition of SYBR Green I dye (Life technologies), gel electrophoresis of resulted products as well as using MX3005P real-time PCR system (Stratagene) which is broadly used for real-time PCR detection of ASFV DNA. The presence of relative fluorescence increase (‘R’-T) during the following minutes of the reaction was considered as positive result. The increment of fluorescent signal gained during the successive PCLSR cycles was proportional to the initial concentration of ASFV DNA within the sample. The conducted PCLSR evaluation using different ASFV genotypes or strains and matrices showed the method is capable to detect DNA of strains belonging to genotype I, II, V, VIII, IX and X (Fig. 4A). The ASFV DNA was detected in bone marrow, blood, serum, spleen, kidneys, lymph nodes, tonsil, lungs or muscle fibers collected from infected pigs or wild boars (Fig. 4B).

### Evaluation of PCLSR using samples from infected wild boars and pigs

**Figure 5:**
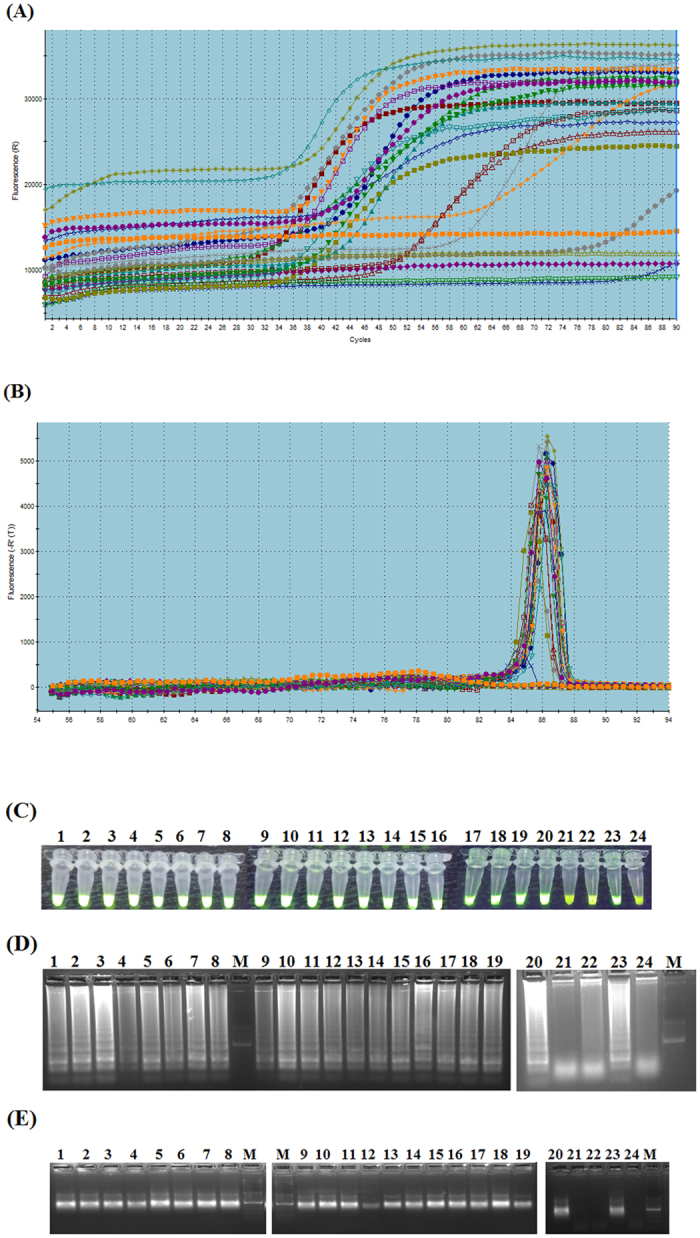
Evaluation of PCLSR. Panel (A) fluorescent curves observed after PCLSR in MX3005 P real-time PCR system (Stratagene). The presence of relative fluorescence increase (‘R’-T) during the following minutes of the reaction was considered as positive result. The curves were observed starting from 32 min. (21^st^ case) up to 78 min. (64^th^ case). Panel (B) melting curve analysis of PCLSR products positive for ASFV’ DNA. The common temperature point for all positive samples is 85.3 °C. Panel (C) PCLSR – assessment of ASFV positive samples after addition of 1 *μ*l of a 1:10 stock dilution of 10,000 x DMSO concentrated SYBR Green I dye (Invitrogen) to the reaction tubes and UV light illumination. Panel (D) gel electrophoresis of (1.5% agarose gel, stained with a SimplySafe solution) (EURx, Gdansk, Poland) of PCLSR products. Visible “ladder-like” pattern in ASFV positive samples. Panel (E) PCR for PCLSR results verification. The PCR was conducted with application of outer-spiral primers. Field samples from 17 ASF cases in wild boar and 3 outbreaks in pigs (lanes 1–20), 21 – blood collected from a non-infected pig, 22 – cDNA extracted from a classical swine fever virus (CSFV) strain Alfort/187, 23 – positive control standard ASFV plasmid containing a p72 gene fragment (7.2 × 10^7^ copies *μ*l^−1^); 24 – cDNA extracted from a porcine reproductive and respiratory syndrome virus (PRRS) strain Lelystad (genotype I). M – molecular length marker GeneRule 100 bp DNA Ladder Plus (Thermo-scientific, Waltham, Massachusetts, USA).

**Figure 6:**
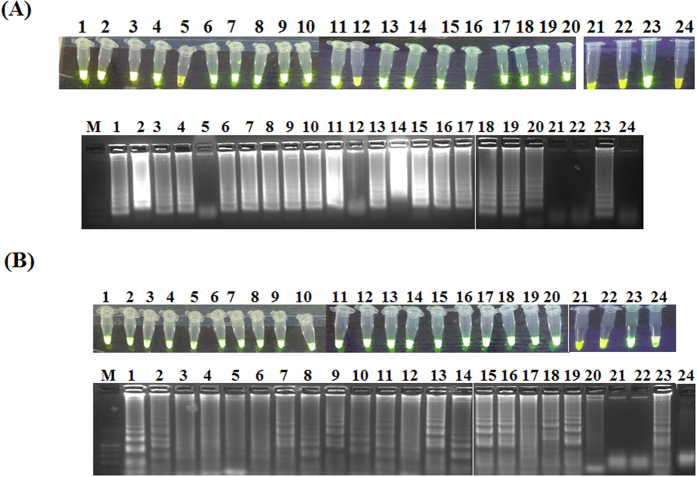
Additional tests using (**A**) LAMP and (**B**) CPA tests using field samples from 17 ASF cases in wild boar and 3 outbreaks in pigs (lanes 1–20). 21 – blood collected from a non-infected pig, 22 – cDNA extracted from a classical swine fever virus (CSFV) strain Alfort/187, 23 – positive control standard ASFV plasmid containing a p72 gene fragment (7.2 × 10^7^ copies *μ*l^−1^); 24 – cDNA extracted from a porcine reproductive and respiratory syndrome virus (PRRS) strain Lelystad (genotype I). M – molecular length marker GeneRuler 100 bp DNA Ladder Plus (Thermo-scientific, Waltham, Massachusetts, USA).

Next, the developed assay was used for amplification of ASFV DNA extracted from blood of wild boars and pigs originating from selected 17 cases and 3 outbreaks (Table 1). This stage of study was conducted for examination of possible application of PCLSR as a simple method for ASFV surveillance. Additionally, a set of negative controls represented by cDNA of CSFV and PRRS was applied to test the specificity of the developed assay. The PCLSR conducted in real-time PCR system resulted in registration of fluorescent curves starting from 32 min (21^st^ case) up to 78 min (64^th^ case) (Fig. 5A). The conducted melting curve analysis showed the common temperature of products for all examined samples reached 85.3 °C (Fig. 5B). All samples containing ASFV DNA from 17 wild boar cases and 3 ASF outbreaks in pigs examined after addition of SYBR Green I showed greenish fluorescence under UV light (Fig. 5C). The conducted electrophoresis of the resulted products in 1.5% agarose gel (Invitrogen) showed the presence of specific ladder-like products in all 20 ASFV positive samples and lack of any product in negative controls (Fig. 5D). In order to confirm the obtained results PCR was conducted using a pair of outer primers. After gel electrophoresis of PCR products in 1.5% agarose gel in all ASFV positive samples the presence of band approximately 185 bp long was observed. In contrast, the presence of products was not observed in specificity controls comprising of nucleic acid extracted from CSFV and PRRS strains (Fig. 5E, lanes 21 and 22), either in negative control of reaction mixture (Fig. 4E, lane 24). Further tests conducted on 55 tissue samples collected from 23 ASF outbreaks in pigs and 22 cases in wild boars showed the ASFV DNA presence in all examined samples (data not presented). Additional tests using LAMP and CPA on field samples from 17 ASF cases in wild boar and 3 outbreaks in pigs showed that samples #5 and #12 were negative in LAMP (Fig. 6A). In case of CPA in spite of remarkable fluorescence the ladder-like products were are weak or invisible in case of samples #3–6, 12 and 20 (Fig. 6B). These results clearly indicate that data obtained by PCLSR are more robust and reliable than LAMP or CPA in comparative results on the specificity of the developed PCLSR and its potential usefulness for on-site detection of ASFV in samples collected from wild boar or pigs.

## Discussion

Diagnosis of numerous infectious animal diseases remains one of the most important tool for early prevention and control^22^,^23^. In case of ASF, diagnosis methods are recommended by OIE as well as EURL in Spain. However, some part of these tools based on PCR methods require advanced laboratory equipment comprising of PCR thermal cycles or more expensive real-time PCR systems^6^,^7^,^8^. In fact, broad application of PCR-based methods during the last 20 years has changed and improved the current diagnosis of ASF. Next, the alternative isothermal amplification methods starting from LAMP in 2001^24^ were implemented into routine detection of many pathogens. Meanwhile, isothermal amplification methods may generate some part of false positive results due to high efficiency of applied polymerases and lack of mechanism of preliminary activation of non-active or blocked enzyme^25^. Therefore, the proper selection or development of novel isothermal technique should especially take into account the possible cross-reactivity of the assay. The developed PCLSR is a novel technique of isothermal amplification that puts together the advantage of traditional PCR or real-time PCR with the relative simplicity of isothermal amplification. Application of external primers with 5′ – ends complementary to the distanced gene of black widow (*Latrodectus hesperus*) alpha-latrotoxin protects the initial steps of reaction. On the basis of the conducted comparison of LAMP, CPA and PCLSR it has been found that PCLSR shares some similarities with these methods but offers higher diagnostic specificity in comparison to the previously described isothermal assays^25^. The cost of all isothermal methods is comparable and reaches approximately 2 EURO per sample. Our previous experience with LAMP, CPA and PSR showed that novel design of PCLSR primers may solve the problem with specificity of isothermal methods. An advantage of this test is the possibility for its on-site ASF diagnosis as a portable assay. The observed diagnostic sensitivity reached 7.2 × 10^2^ copies of plasmid containing a fragment of p72 gene which was below the value of 7.2 copies reached by CPA or real-time PCR^14^. Similarly to previously described CPA, this method might be used by veterinary practitioners, veterinary officers or hunters. The isothermal methods including PCLSR seem to be a future alternative for PCR and real-time PCR assays but are not recommended by OIE yet. Hopefully, future application of PCLSR may assist in development of better biosecurity measures in terms of transportation, destruction of ASFV infectious material storage.

## Methods

### Samples origin

All samples originating from pigs and wild boars used in this study were collected by the official county veterinary officers and certified hunters in accordance with strict regulations of the Chief Veterinary Officer and ‘Biosecurity Programme aimed at prevention of African Swine Fever spread for the years 2015–2018’ (Journal of Laws, 2014–2016, item no. 679) approved by the Polish Ministry of Agriculture and Rural Development. All experimental protocols used in this study for detection of ASFV were approved by the National Veterinary Research Institute in Pulawy, Poland.

### Clinical samples

Clinical samples of blood were collected from selected 17 ASFV cases (5^th^, 16^th^, 17^th^, 21^st^, 36^th^, 39^th^ (a), 39^th^ (b), 42^nd^, 44^th^, 60^th^, 61^st^, 64^th^, 66^th^, 69^th^, 72^nd^, 73^rd^, 75^th^) and 3 outbreaks (1^st^–3^rd^) in pigs. The collected samples were sent to the National Veterinary Research Institute in Pulawy (NVRI). Additional set of 55 samples collected from 23 ASF outbreaks in pigs and 23 cases in wild boars has been tested. The samples were collected between February 2014 and December 2016. All samples were first tested using OIE and European Reference Laboratory (EURL) recommended techniques including UPL real-time PCR^8^, ELISA or a confirmatory immunoperoxidase test (IPT)^5^.

### Positive and negative controls

The negative DNA control samples used for the PSCLR were extracted from the peripheral blood of healthy pigs and wild boars. Additionally a panel of cDNA extracted from classical swine fever virus (CSFV) strain Alfort/187, porcine reproductive and respiratory syndrome virus (PRRS) strain Lelystad (genotype I) and field porcine epidemic diarrhea virus (PEDV-NVRI2015) were used. These samples were then tested using PCLSR. A set of ASFV strains belonging to the genotype I (CV98, BF07, E70), genotype II (Arm07, LT14/1490, Ukr12/Zapo, Estonia 1, Estonia 2, Estonia 3, Estonia 4, Estonia 5, Estonia 6, Estonia 7), genotype V (Moz64), genotype VIII (Malawi), genotype IX (Ken06.Bus), genotype X (Ken08K.2/1, Ug64) have also been used. A panel of different matrices represented by bone marrow, blood of pig, serum of pig, blood of wild boar, spleen, kidneys, lymph nodes, tonsi1, lungs, muscle fibers have also been tested.

### Primer design

A set of three specific primers for PCLSR was designed manually, based on the p72 ASFV gene sequence of a Georgia 2007/1 isolate (Accession number: FR682468.1) and black widow (*Latrodectus hesperus*) alpha-latrotoxin (Genbank accession: KF751511.1). The primer sequences were verified with GenBank database using BLAST algorithm (www.ncbi.nlm.nih.gov/BLAST) to confirm their specificity to the target sequences.

### PCLSR optimization

The PCLSR temperature and duration was optimized at various temperatures, ranging from 55.0 °C to 66.0 °C and from 30 to 90 min, respectively. The assay volume was 15 *μ*l, which contained, 7.5 *μ*l of Isothermal Mastermix (OptiGene, Horsham, West Sussex, United Kingdom) 10–40 pmol of cross-linking primer (Link AB) 10–40 pmol of each outer primers, 1 *μ*l of standard plasmid, containing a p72 gene or DNA extracted from blood of infected wild boars or pigs. After reaction, 1 *μ*l of a 1:10 stock dilution of 10,000 x DMSO concentrated SYBR Green I dye (Invitrogen) was added to each reaction tube. The results were read under UV illumination. The presence of greenish fluorescence in positive samples indicated the presence of ASFV DNA. The PCLSR sensitivity was evaluated using serial 10-fold dilutions of a standard ASFV plasmid with p72 gene fragment (from 7.2 × 10^7^ copies to 0.07 copies, *μ*l^−1^). The resulted PCLSR products separated in 1.5% agarose gels, stained with a SimplySafe solution (0.5 *μ*g ml^−1^) (EURx, Gdansk, Poland) under a voltage of 100 V/50 min, to detect “ladder-like” pattern of products. Parallelly, the PCLSR was conducted in MX3005P real-time PCR system (Stratagene). The presence of relative fluorescence increase (‘R’-T) during the following minutes of the reaction was considered as a positive result.

### Purification and sequencing of PCLSR products

The PCLSR products were purified using Nucleo-Spin^®^ Gel and PCR Clean up (Macherey-Nagel, Düren, Germany) sequenced in both forward and backward directions on GS FLX/Titanium sequencer (Roche, Branford, Connecticut, USA) by Genomed S.A. (Warsaw, Poland). The sequences were compared with reference records as Georgia 2007/1 (Accession number: FR682468.1) and Odintsovo 2/2014 (Accession number: KP843857.1).

### DNA extraction

The DNA from the reference virus stock as well as the whole blood from wild boars and pigs was extracted using High Pure PCR Template Preparation Kit, accordingly to the manufacturer’s procedure (Roche Diagnostics, Basel, Switzerland). The extracted DNA was stored at −20 °C for further testing.

### PCR

The PCR for PCLSR results verification was performed in 25 μl final volume using outer PCLSR primers accordingly to the procedure of MyTaq HS DNA Polymerase kit (Bioline, Gdansk, Poland). The reaction mixture contained: 12.5 μl of MyTaq™ HS DNA Polymerase, 9.5 μl PCR-grade water, 1 μl of each primer and 1 μl (~200 ng) template DNA. The primer concentration was 40 pM of each primer. The obtained PCR products were subjected to electrophoresis in 1.5% agarose gels under voltage of 100 V/50 min. The gels were stained by addition of 5 μl of SimplySafe solution as for PCLSR product separation. The length of PCR products were estimated on the basis of 100 bp DNA Ladder Plus GeneRuler (Thermo-scientific, Waltham, Massachusetts, USA). All PCLSR were replicated to verify reproducibility.

### LAMP

LAMP has been conducted using previously described primers^13^ in 15 *μ*l reaction volume using the following concentration of reagents: 7.5 *μ*l of Isothermal Mastermix 50 pmol of forward and backward inner primer (FIP and BIP), 10 pmol of outer primers (F3 and B3) and 25 pmol of loop primers (LF and LB), 1 *μ*l of standard plasmid, containing a p72 gene. After incubation, 1 *μ*l of a 1:10 stock dilution of 10,000 x DMSO concentrated SYBR Green I dye (Invitrogen) was added to the reaction vessel. The results were registered under UV illumination, to detect fluorescence.

### CPA

The CPA was conducted accordingly to the previously described protocol^14^,^26^. Briefly, the reaction was conducted at 56.2 °C for 45 min. The final CPA assay volume was 15 *μ*l, and contained 7.5 *μ*l of Isothermal Mastermix 20 pmol of cross-primer (1 s) 10 pmol of each inner primer (2a and 3a) 5 pmol of each outer primer 4 s and 5a and 1 *μ*l of standard plasmid, containing a p72 gene. Results detection was conducted identically to PCLSR and LAMP techniques.

## Additional Information

**How to cite this article:** Woźniakowski, G. *et al*. Polymerase cross-linking spiral reaction (PCLSR) for detection of African swine fever virus (ASFV) in pigs and wild boars. *Sci. Rep.* **7**, 42903; doi: 10.1038/srep42903 (2017).

**Publisher's note:** Springer Nature remains neutral with regard to jurisdictional claims in published maps and institutional affiliations.

## Figures and Tables

**Table 1:** The origin of samples used for PCLSR examination.

Case (C) or (O) outbreak number	Localisation (nearest town)	Localisation (county)	Collection date	Sample origin (D-dead, H-hunted wild boar)/tissue
C5	Słoja	Sokółka	24.06.2014	D/spleen
C16	Straszewo	Białystok	22.09.2014	H/blood
C17	Nowosady Kolonia	Białystok	04.10.2014	D/blood and kidney
C21	Wiejki	Białystok	21.11.2014	D/blood and spleen
C36	Wierzchlesie	Białystok	13.02.2015	D/bone
C39	kolonia Bachury	Białystok	02.03.2015	D/bone
C39	kolonia Bachury	Białystok	02.03.2015	D/bone
C42	Bielewicze	Białystok	15.03.2015	D/blood and spleen
C44	Kolonia Cisówka	Białystok	25.03.2015	D/bone
C60	Dzierniakowo	Białystok	13.05.2015	D/lung
C61	Nowosady	Białystok	20.05.2015	D/spleen
C64	Planty	Białystok	1.06.2015	D/bone
C66	Babia Góra	Hajnówka	25.06.2015	D/bone
C69	Załuki	Białystok	02.07.2015	D/bone
C72	Szudziałowo	Sokółka	14.07.2015	D/bone
C73	Białowieża	Hajnówka	20.07.2015	H/blood
C75	Pogorzelce	Hajnówka	13.08.2015	D/bone
O1	Zielona	Białystok	19.07.2014	D/spleen
O2	Józefowo	Białystok	06.08.2014	D/spleen
O3	Puciłki	Sokółka	27.01.2015	D/spleen

The number of case (C) or outbreak (O) has been presented.
